# Prognostic signature related to the immune environment of oral squamous cell carcinoma

**DOI:** 10.1515/biol-2022-0467

**Published:** 2022-09-14

**Authors:** Yingjie Hua, Xuehui Sun, Kefeng Luan, Changlei Wang

**Affiliations:** Department of Stomatology, Affiliated Hospital of Weifang Medical University, 2428 Yuhe Road, Kuiwen District, Weifang City, Shandong Province, 261041, China

**Keywords:** oral cancer, subtype, risk score, prognosis, survival

## Abstract

Oral squamous cell carcinoma (OSCC) prognosis remains poor. Here we aimed to identify an effective prognostic signature for predicting the survival of patients with OSCC. Gene-expression and clinical data were obtained from the Cancer Genome Atlas database. Immune microenvironment-associated genes were identified using bioinformatics. Subtype and risk-score analyses were performed for these genes. Kaplan–Meier analysis and immune cell infiltration level were explored in different subtypes and risk-score groups. The prognostic ability, independent prognosis, and clinical features of the risk score were assessed. Furthermore, immunotherapy response based on the risk score was explored. Finally, a conjoint analysis of the subtype and risk-score groups was performed to determine the best prognostic combination. We found 11 potential prognostic genes and constructed a risk-score model. The subtype cluster 2 and a high-risk group showed the worst overall survival; differences in survival status might be due to the different immune cell infiltration levels. The risk score showed good performance, independent prognostic value, and valuable clinical application. Higher risk scores showed higher Tumor Immune Dysfunction and Exclusion scores, indicating that patients with a high-risk score were less likely to benefit from immunotherapy. Finally, conjoint analysis for the subgroups and risk groups showed the best predictive ability.

## 1 Introduction

Head and neck squamous cell carcinoma (HNSCC) is a heterogeneous malignant tumor that arises from the squamous epithelium of the oral cavity, oropharynx, larynx, and hypopharynx [1]. Oral squamous cell carcinoma (OSCC) is the main subtype of HNSCC, accounting for approximately 30–40% of the cases [2]. Traditional risk factors for OSCC include consumption of tobacco and alcohol as well as human papillomavirus infection [3]. The prognosis of patients with OSCC is relatively poor, with only an approximately 50% 5 year survival rate [4]. About 10–30% of the patients with advanced-stage OSCC present locoregional recurrences, and 15–25% manifest distant metastases [5]. Thus, the identification of relevant prognostic biomarkers is necessary for decision-making to improve patient outcomes. Many prognosis-related gene biomarkers have been identified; however, they are still under molecular exploration, and relevant clinical verification is lacking.

Currently, the treatment for OSCC focuses on surgery accompanied by chemotherapy or radiotherapy. Recently, immunotherapy has achieved more favorable outcomes, as some patients with HNSCC demonstrate improved clinical survival following the blockade of PD-1 and PD-L1 [6]. Numerous studies have found that OSCC is correlated with the immune microenvironment; however, the specific mechanism remains obscure. To date, several studies have found the important regulatory roles of immune cells and stromal cells in the development of tumors. Improved overall survival (OS) of patients with OSCC is associated with high levels of CD8+ T-cell infiltration [7,8]. Additionally, regulatory T-cells participate in the creation of an immunosuppressive environment in HNSCC [9] and OSCC [10]. Cillo et al. also emphasized the important roles of CD8+ T-cells and regulatory T-cells in HNSCC [11]. Therefore, the immune microenvironment plays a significant role in tumor development.

Although immunotherapy has achieved considerable results, some patients and tumor types cannot benefit from immunotherapy [11]. In addition, OSCC, like many other types of cancer, is associated with genomic alterations [5]. Therefore, it is necessary to identify distinctive OSCC subgroups for the development of more individualized treatments. Bioinformatics has been used in the identification of several molecular markers and the construction of promising prognostic models [12,13,14]. The widespread use of bioinformatics tools facilitates the understanding of numerous diseases. Therefore, we aimed to use bioinformatics to identify a useful prognostic signature related to the tumor immune microenvironment in OSCC.

## 2 Methods

### 2.1 Data resource

**Figure 1:**
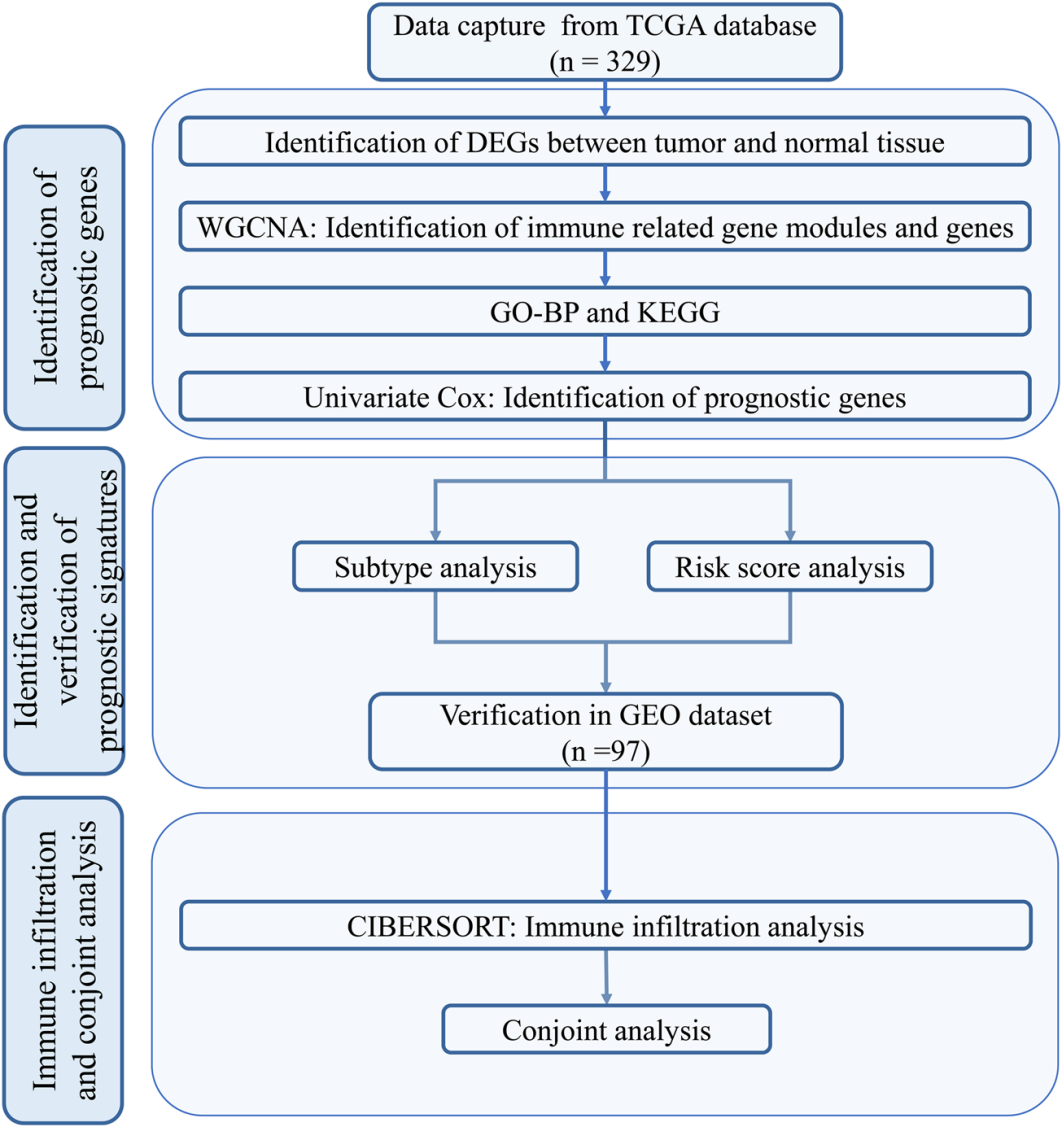
Study design. OSCC, oral squamous cell carcinoma; DEGs, differentially expressed genes; WGCNA, weighted gene co-expression network analysis; GO-BP, gene ontology–biological process; KEGG, Kyoto encyclopedia of genes and genomes; LASSO, least absolute shrinkage and selection operator; ROC, receiver operator characteristic.

Gene expression data and corresponding clinical data for HNSC in The Cancer Genome Atlas (TCGA) portal were downloaded from the UCSC Xena database (https://xena.ucsc.edu/). The expression data were normalized with log2 (fragments per kilobase of exon per million mapped fragments [FPKM] + 1). Data from clinical samples of the oral cavity (lip, tongue, alveoli, floor of mouth, ridge, buccal mucosa, hard palate, and oral cavity) were used for further analysis, while samples from other anatomic sites (hypopharynx, larynx, oropharynx, and tonsil) were excluded. After removing samples with incomplete OS data, 329 OSCC tumor samples with a detailed follow-up period were included for subsequent analysis. Clinical data from the GSE41613 [15] dataset were downloaded from the Gene Expression Omnibus (GEO) database (https://www.ncbi.nlm.nih.gov/). Detailed information on the TCGA-OSCC and GEO datasets is shown in Tables 1 and 2. A total of 97 patients with OSCC with a detailed follow-up time were included in this study. The study design is illustrated in Figure 1.

**Table 1:** Information of TCGA-OSCC datasets

Characteristics	Alive (*N* = 188)	Dead (*N* = 154)	Total (*N* = 342)
OS time (years)			
Mean value ± SD	2.89 ± 2.21	1.89 ± 2.40	2.44 ± 2.35
Median [min–max]	2.33 [0.03–15.01]	1.11 [0.03–14.12]	1.76 [0.03–15.01]
Age			
Mean value ± SD	59.93 ± 12.36	63.42 ± 13.11	61.50 ± 12.80
Median [min–max]	60.00 [19.00–85.00]	63.00 [24.00–90.00]	61.00 [19.00–90.00]
Clinical_M			
M0	171 (50.44%)	152 (44.84%)	323 (95.28%)
M1	1 (0.29%)	1 (0.29%)	2 (0.59%)
MX	13 (3.83%)	1 (0.29%)	14 (4.13%)
Clinical_N			
N0	96 (28.32%)	75 (22.12%)	171 (50.44%)
N1	30 (8.85%)	30 (8.85%)	60 (17.70%)
N2	6 (1.77%)	5 (1.47%)	11 (3.24%)
N2a	5 (1.47%)	4 (1.18%)	9 (2.65%)
N2b	24 (7.08%)	22 (6.49%)	46 (13.57%)
N2c	12 (3.54%)	14 (4.13%)	26 (7.67%)
N3	3 (0.88%)	2 (0.59%)	5 (1.47%)
NX	9 (2.65%)	2 (0.59%)	11 (3.24%)
Clinical_T			
T1	14 (4.13%)	7 (2.06%)	21 (6.19%)
T2	62 (18.29%)	45 (13.27%)	107 (31.56%)
T3	43 (12.68%)	42 (12.39%)	85 (25.07%)
T4	7 (2.06%)	11 (3.24%)	18 (5.31%)
T4a	50 (14.75%)	47 (13.86%)	97 (28.61%)
T4b	1 (0.29%)	2 (0.59%)	3 (0.88%)
TX	8 (2.36%)	0 (0.0e + 0%)	8 (2.36%)
Clinical_stage			
I	7 (2.11%)	5 (1.51%)	12 (3.61%)
II	44 (13.25%)	36 (10.84%)	80 (24.10%)
III	40 (12.05%)	30 (9.04%)	70 (21.08%)
IVA	82 (24.70%)	78 (23.49%)	160 (48.19%)
IVB	4 (1.20%)	3 (0.90%)	7 (2.11%)
IVC	1 (0.30%)	2 (0.60%)	3 (0.90%)
Gender			
Female	53 (15.50%)	53 (15.50%)	106 (30.99%)
Male	135 (39.47%)	101 (29.53%)	236 (69.01%)
Histologic_grade			
G1	31 (9.14%)	22 (6.49%)	53 (15.63%)
G2	114 (33.63%)	91 (26.84%)	205 (60.47%)
G3	33 (9.73%)	38 (11.21%)	71 (20.94%)
G4	4 (1.18%)	0 (0%)	4 (1.18%)
GX	3 (0.88%)	3 (0.88%)	6 (1.77%)

**Table 2:** Information of GSE41613

Characteristics	Alive (*N* = 46)	Dead (*N* = 51)	Total (*N* = 97)
OS time (months)			
Mean value ± SD	66.57 ± 8.92	23.89 ± 19.92	44.13 ± 26.52
Median [min–max]	65.34 [52.60–85.03]	18.40 [0.46–78.29]	54.41 [0.46–85.03]
Age			
Mean value ± SD	53.98 ± 8.92	54.43 ± 6.39	54.22 ± 7.66
Median [min–max]	60.00 [19.00–60.00]	55.00 [39.00–60.00]	55.00 [19.00–60.00]
Gender			
Female	15 (15.46%)	16 (16.49%)	31 (31.96%)
Male	31 (31.96%)	35 (36.08%)	66 (68.04%)
Stage			
I/II	30 (30.93%)	11 (11.34%)	41 (42.27%)
III/IV	16 (16.49%)	40 (41.24%)	56 (57.73%)

### 2.2 Calculation of immune and stromal scores

The estimate package [16] in R3.6.1 was used to calculate the immune and stromal scores based on the OSCC data from TCGA. Wilcoxon tests were conducted to compare the differential infiltration of immune and stromal cells between OSCC tumors and normal adjacent tissue samples.

### 2.3 Selection and analysis of hub module genes

Differentially expressed genes (DEGs) between tumor and normal samples from TCGA-OSCC were identified using the Limma package [17] in R3.6.1. The selection threshold was defined as false discovery rate (FDR) < 0.05, and |log2 fold change (FC)| > 1. Hierarchical clustering of DEG expression was performed, and the results were visualized in heat maps using pheatmap (version 1.0.8) in R3.6.1 [18]. The DEGs were subjected to weighted gene co-expression network analysis (WGCNA) [19] to select the differential immune microenvironment-related gene set as the module genes. Gene Ontology–Biological Process (GO-BP) and Kyoto Encyclopedia of Genes and Genomes (KEGG) analyses were conducted based on the module genes to obtain significantly related biological processes and KEGG pathways (with *p* < 0.05) in DAVID 6.8 [20] (https://david.ncifcrf.gov/).

### 2.4 Selection of prognosis-related genes

Univariate Cox analysis of the survival package [21] in R3.1.6 was conducted to select prognostic genes that were significantly associated with survival (*p* < 0.05).

### 2.5 Analysis of OSCC subtype

Based on the prognostic genes, tumor samples from TCGA-OSCC were grouped into different subtypes using ConsensusClusterPlus [22] (version 1.54.0) in R3.6.1. Subsequently, Kaplan–Meier curve analysis in the survival package [21] was performed to evaluate the survival status of the different subtypes. Finally, the proportion of 22 immune cells was calculated using CIBERSORT based on TCGA samples, and the differential infiltration of immune cells was analyzed between the different OSCC subtypes.

### 2.6 Construction and evaluation of the prognostic risk model

To identify the optimal prognostic genes, the prognostic genes were analyzed using the least absolute shrinkage and selection operator (LASSO) Cox regression in the lars package [23] in R3.6.1. Subsequently, the prognostic model was constructed as follows: Risk score = ∑*β*_genes_ × Exp_genes_, where *β*_genes_ refers to the LASSO coefficient of genes and Exp_genes_ refers to the expression level of genes in TCGA. The samples in TCGA and GSE41613 were grouped into high- and low-risk groups, with their median risk score as the threshold. The Kaplan–Meier curve [21] was used to explore the differential survival between the two risk groups. Receiver operating characteristic (ROC) curves and area under the curve (AUC) values were used to assess the predictive ability of the risk model. To determine the independent prognostic value of the risk score, clinical factors such as age, sex, stage, and risk score were combined into univariate and multivariate Cox regression analyses. Independent prognostic elements were selected under *p* < 0.05, and forest plots were visualized.

### 2.7 Immune microenvironment and immunotherapy response analysis

The proportion of 22 immune cells was calculated using CIBERSORT based on TCGA samples, and differential infiltration of immune cells was analyzed between different risk groups. Furthermore, the correlation between the immune cells with significantly different infiltration and the optimal prognostic genes was analyzed. Next, patient response to immune checkpoint inhibitor (ICI) therapy was predicted in the Tumor Immune Dysfunction and Exclusion (TIDE) database (http://tide.dfci.harvard.edu/). Individual responses to immunotherapy were measured using the TIDE score. TIDE is a computational method that models two primary mechanisms of tumor immune escape, T-cell dysfunction, and T-cell exclusion, which could be used to predict the ICI response in patients with cancer [24]. A high TIDE score indicates a higher potential for tumor immune evasion and reduced chances of benefiting from ICI therapies.

### 2.8 Clinical prognostic value of the risk model

To investigate the predictive value of the risk score in clinical applications, we further analyzed the performance of different risk groups in patients divided by gender, tumor-node-metastasis (TNM) stage, and grade. Finally, to explore a more effective prediction method for patients with OSCC, a conjoint analysis combining the two subtypes and the two risk groups was conducted. The integrated and risk-score groups were combined into three new groups: low-C1 (low-risk group and cluster 1), low-C2/high C1 ([low-risk group and cluster 2] or [high-risk group and cluster 1]), and high-C2 (high-risk group and cluster 2). The Kaplan–Meier curve [21] was used to evaluate the survival of the new groups. Subsequently, the C-index of the clinical factors (including age, gender, TNM stage, and grade), subtype groups, risk-score groups, and combined groups were calculated.

All the abbreviations are listed in Table 3.

**Table 3:** Abbreviations and their full name

Abbreviation	Full name
HNSCC	Head and neck squamous cell carcinoma
OSCC	Oral squamous cell carcinoma
HPV	Human papillomavirus
TCGA	The Cancer Genome Atlas
GEO	Gene Expression Omnibus
DEGs	Differentially expressed genes
WGCNA	Weighted gene co-expression network analysis
GO-BP	Gene Ontology–Biological Process
KEGG	Kyoto Encyclopedia of Genes and Genomes
LASSO	Least absolute shrinkage and selection operator
ROC	Receiver operating characteristic
TIDE	Tumor Immune Dysfunction and Exclusion
ICI	Immune checkpoint inhibitors

## 3 Results

### 3.1 Differential stromal and immune scores between tumor and normal tissue

**Figure 2:**
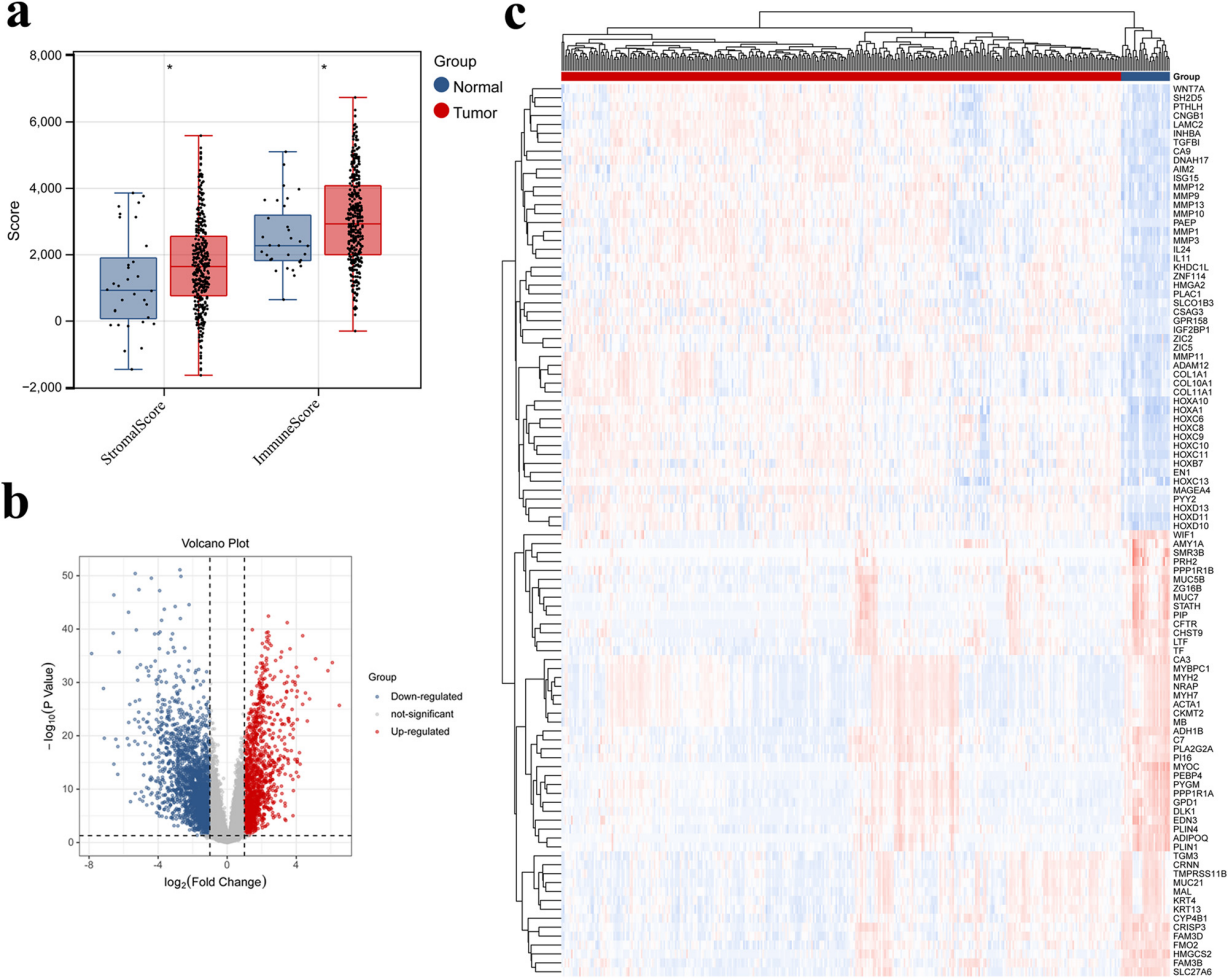
Selection of DEGs. (a) The differences in immune/stromal score between tumor and normal tissue. (b) Volcano plot identifying DEGs. Red and blue points refer to significantly up and downregulated genes, respectively; the horizontal dotted line represents *p* = 0.05; two vertical dotted lines represent |log2FC| = 0.5. (c) Heat plot of the top 50 upregulated and downregulated DEGs.

An estimate algorithm was used to calculate the immune and stromal scores based on the TCGA database. The differences in the immune and stromal scores between OSCC and normal tissues were compared (Figure 2a). The boxplots showed that both the stromal and immune scores in tumor tissues were remarkably higher than those in normal tissue.

### 3.2 Selection of hub model genes

**Figure 3:**
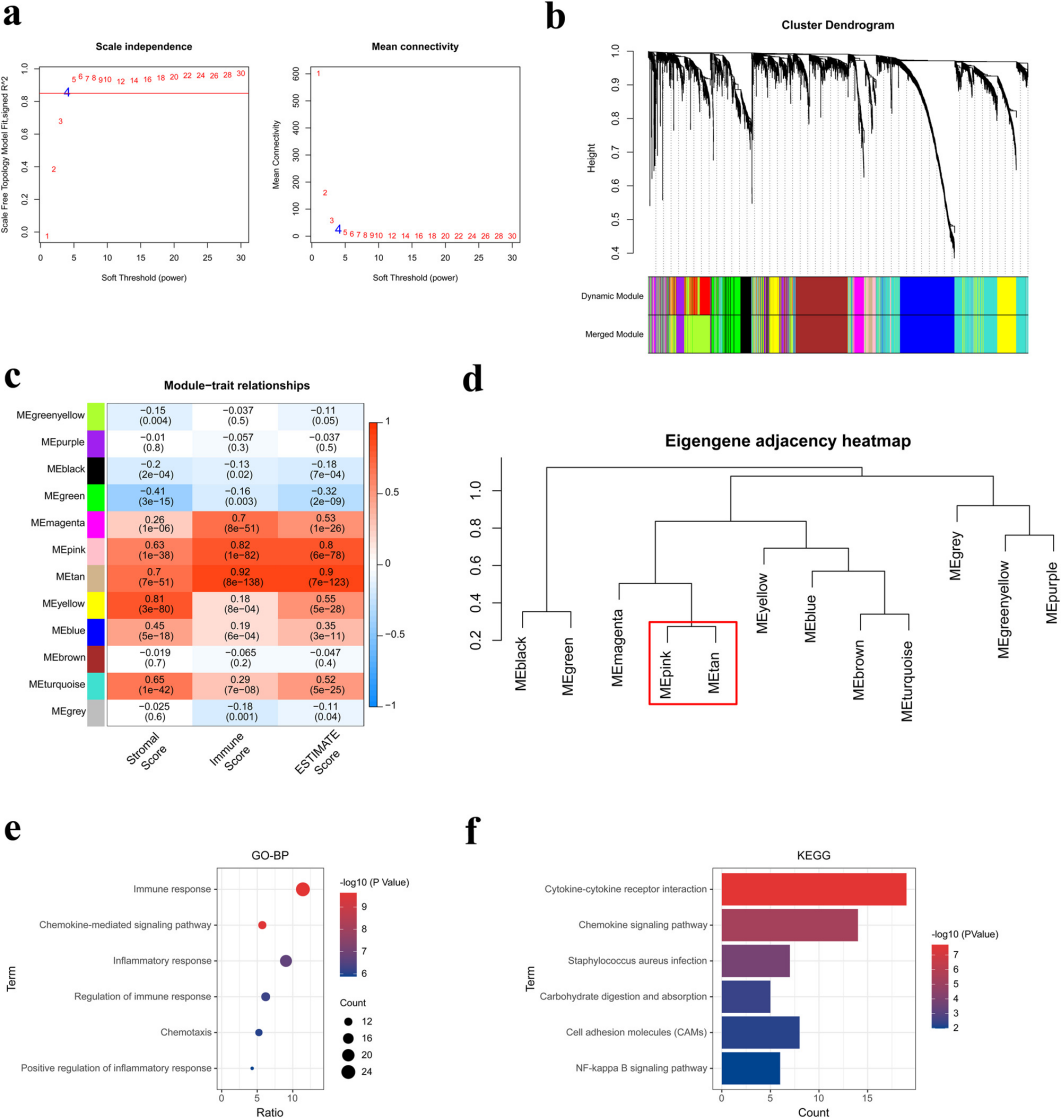
Selection of hub model genes. (a) Selection of the soft-thresholding powers in WGCNA analysis. The left panel shows the scale-free fit index vs the soft-thresholding power. The right panel displays the mean connectivity vs the soft-thresholding power. Power 4 was chosen because the fit index curve flattened out upon reaching a high value (>0.85). (b) Dendrogram of all DEGs clustered based on the measurement of dissimilarity. Different colors refer to different modules. (c) Heat plot of the correlation between different modules and clinical features. (d) Dendrogram of the relationship of the modules. (e) Analysis of GO-BP. The size of the dot represents the number of genes. The horizontal axis represents the gene proportion, and the vertical axis represents the item name. (f) Signaling pathways in KEGG. The horizontal axis represents the number of genes, and the vertical axis represents the item name.

The Limma package was used to explore the differential expression levels of genes between OSCC tumor and normal groups, and the threshold was defined as FDR < 0.05 and |log2FC| > 1. A total of 3,783 eligible DEGs were identified. Volcano and heat plots are shown in Figure 2b and c, respectively. The 3,783 DEGs were included in the WGCNA analysis and divided into 12 modules (Figures 3a and b). The correlation between each module and clinical features was calculated (Figure 3c), and the most relevant modules with clinical relevance (tan and pink) were selected as the key modules. Sixty and 161 genes were identified in the tan and pink modules, respectively. Further analysis suggested that the tan module was related to the pink module (Figure 3d). Thus, we generated them into a single large model containing 221 genes in total.

### 3.3 Function analysis of hub model genes

Biological function annotation and the KEGG signaling pathway of the 221 hub genes were analyzed in DAVID. In total, 71 significantly relevant GO-BP and 13 KEGG pathways were identified at *p* < 0.05. The top six GO-BP and KEGG pathways are shown in Figure 3e and f, respectively. Significantly, these biological functions were closely correlated with immune response, inflammatory response, and chemokine-related pathways.

### 3.4 Analysis of OSCC subtype

**Figure 4:**
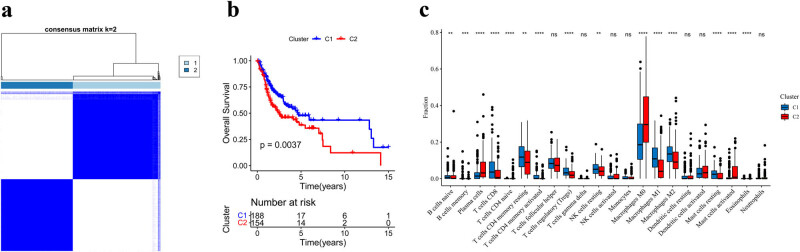
Analysis of subtypes. (a) All samples are grouped into two subtypes: cluster 1 (C1, 188 samples) and cluster 2 (C2, 154 samples). (b) Kaplan–Meier curve of the two clusters. (c) The immune cell infiltration analysis between the two clusters. C1, cluster 1; C2, cluster 2.

Based on the 221 hub genes mentioned above, a univariate Cox analysis was conducted to identify the prognostic genes. A total of 34 genes were significantly associated with prognosis. Subsequently, based on the 34 prognostic genes, all TCGA-OSCC samples were grouped into two subtypes (Figure 4a): cluster 1 (188 samples) and cluster 2 (154 samples). Kaplan–Meier analysis was conducted to evaluate the prognosis of the different subtypes. The curve showed that cluster 2 had a less favorable survival outcome than cluster 1 (Figure 4b). To explore the differential immune features between the two clusters, the CIBERSORT algorithm was used to evaluate the infiltration level of immune cells, and the differences between the two clusters were analyzed (Figure 4c). Finally, 15 differentially expressed immune cell lines were selected. Cluster 2 showed lower levels of immune cell infiltration, including CD8+ T-cells, activated CD4 + memory T-cells, and regulatory T-cells.

### 3.5 Construction and evaluation of the prognostic risk model

**Figure 5:**
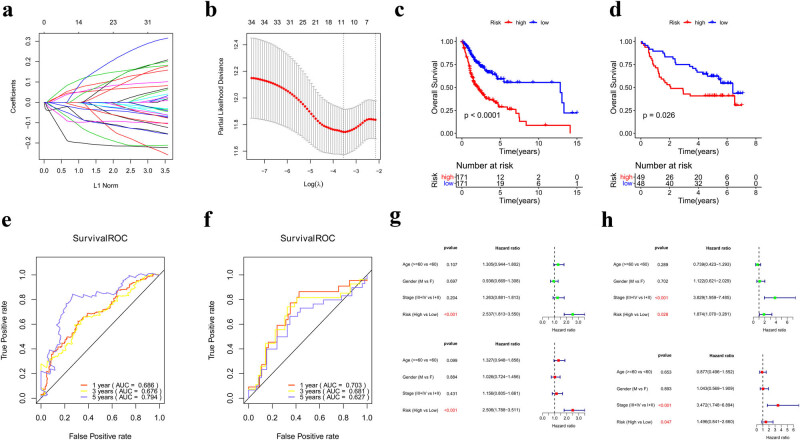
Construction and evaluation of the risk-score model. (a) LASSO coefficient distribution of 34 genes. (b) Likelihood of bias in LASSO coefficient distribution. Left and right vertical dashed lines representing lambda.min and lambda.1se, respectively. A total of 11 prognostic genes were identified. (c and d) Kaplan–Meier curve in the TCGA (c) and GSE41613 datasets (d). (e and f) ROC curve in the TCGA (e) and GSE41613 datasets (f). (g) Univariate and multivariate Cox analysis conducted in the TCGA. (h) Univariate and multivariate Cox analysis conducted in the GSE41613.

The same 34 prognostic genes, after univariate Cox analysis, were also included in the LASSO algorithm to filter the optimal prognostic genes (Tables S1 and S2; Figure 5a and b). In total, 11 optimal prognostic genes were identified for subsequent analysis (Table 4). According to the LASSO coefficient and the gene expression level in TCGA, the risk-score model was constructed as follows: Risk score = (−0.0215) × Exp *CTLA4* + (−0.0677) × Exp *TNFRSF4* + (−0.0487) × Exp *KLHL6* + (−0.0878) × Exp *HAO2* + (−0.1814) × Exp *OSR2* + (0.0432) × Exp *ZFP42* + (0.0803) × Exp *RTN4R* + (−0.0189) × Exp *FCGBP* + (0.0513) × Exp *IGF2BP2* + (−0.0023) × Exp *KCNA2* + (0.0370) × Exp *FST*. Subsequently, the risk scores of the samples in the TCGA and GSE41613 datasets were calculated. All the samples were grouped into high- and low-risk groups, using the median risk-score as the threshold. Kaplan–Meier analysis was performed in TCGA to evaluate the OS of the two risk groups. The curves showed higher survival rates in the low-risk group than in the high-risk group (Figure 5c). ROC analysis demonstrated that the established risk-score model in this study possessed a promising predictive ability for patients with OSCC at years 1, 3, and 5, with AUCs of 0.686, 0.676, and 0.794, respectively (Figure 5d). Kaplan–Meier and ROC analyses conducted for GSE41613 also strengthened the results (Figure 5e and f). Furthermore, univariate and multivariate Cox analyses were conducted to validate independent prognostic factors. Combining the clinical information and risk score, the risk score was confirmed to be an independent predicting element (Figure 5g and h).

**Table 4:** Univariate Cox regression and LASSO analysis of 11 genes

Gene	Univariate COX regression		LASSO coefficient
	HR (95% CI)	*p* value	
CTLA4	0.8473 (0.7671–0.9357)	0.0011	−0.0215
TNFRSF4	0.8158 (0.7136–0.9327)	0.0029	−0.0677
KLHL6	0.8316 (0.7335–0.9427)	0.0039	−0.0487
HAO2	0.7287 (0.5824–0.9117)	0.0056	−0.0878
OSR2	0.8146 (0.7044–0.9420)	0.0057	−0.1814
ZFP42	1.0873 (1.0226–1.1560)	0.0075	0.0432
RTN4R	1.2464 (1.0604–1.4650)	0.0076	0.0803
FCGBP	0.9041 (0.8371–0.9766)	0.0104	−0.0189
IGF2BP2	1.1934 (1.0350–1.3760)	0.0149	0.0513
KCNA2	0.8383 (0.7214–0.9741)	0.0213	−0.0023
FST	1.1147 (1.0079–1.2328)	0.0346	0.0370

### 3.6 Correlation of risk core and potential prognostic genes with immune cells

**Figure 6:**
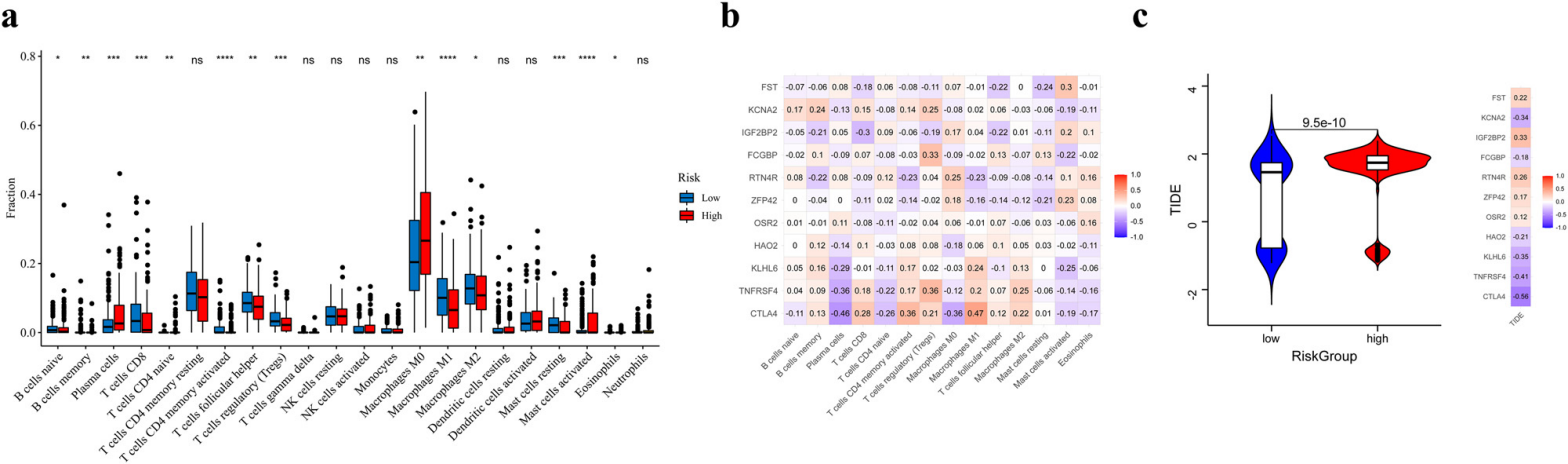
The immune cell infiltration analysis for the risk score. (a) The immune cell infiltration analysis between the two risk groups: high-risk group and low-risk group. (b) The correlation of 14 significantly different immune cells and 11 potential prognostic genes in the risk model. (c) TIDE score analysis with the risk score.

Differential immune cell infiltration between the two risk groups was also analyzed, and the results showed that the infiltration of 14 immune cells significantly differed between the two groups (Figure 6a). Compared with the high-risk group, the low-risk group showed higher T- and B-cell infiltration. Furthermore, we investigated the correlation of the 11 potential prognostic genes with the 14 immune cells (Figure 6b). The results showed that these genes were highly correlated with these immune cell infiltration levels.

The TIDE score was higher in the high-risk group than in the low-risk group (Figure 6c), indicating that patients in the high-risk group were more likely to experience tumor immune evasion and less likely to benefit from ICI therapies. Furthermore, the 11 potential prognostic genes were highly related to the TIDE score, demonstrating their potential predicting value in OSCC.

### 3.7 Prognostic value of the risk score in clinical applications

**Figure 7:**
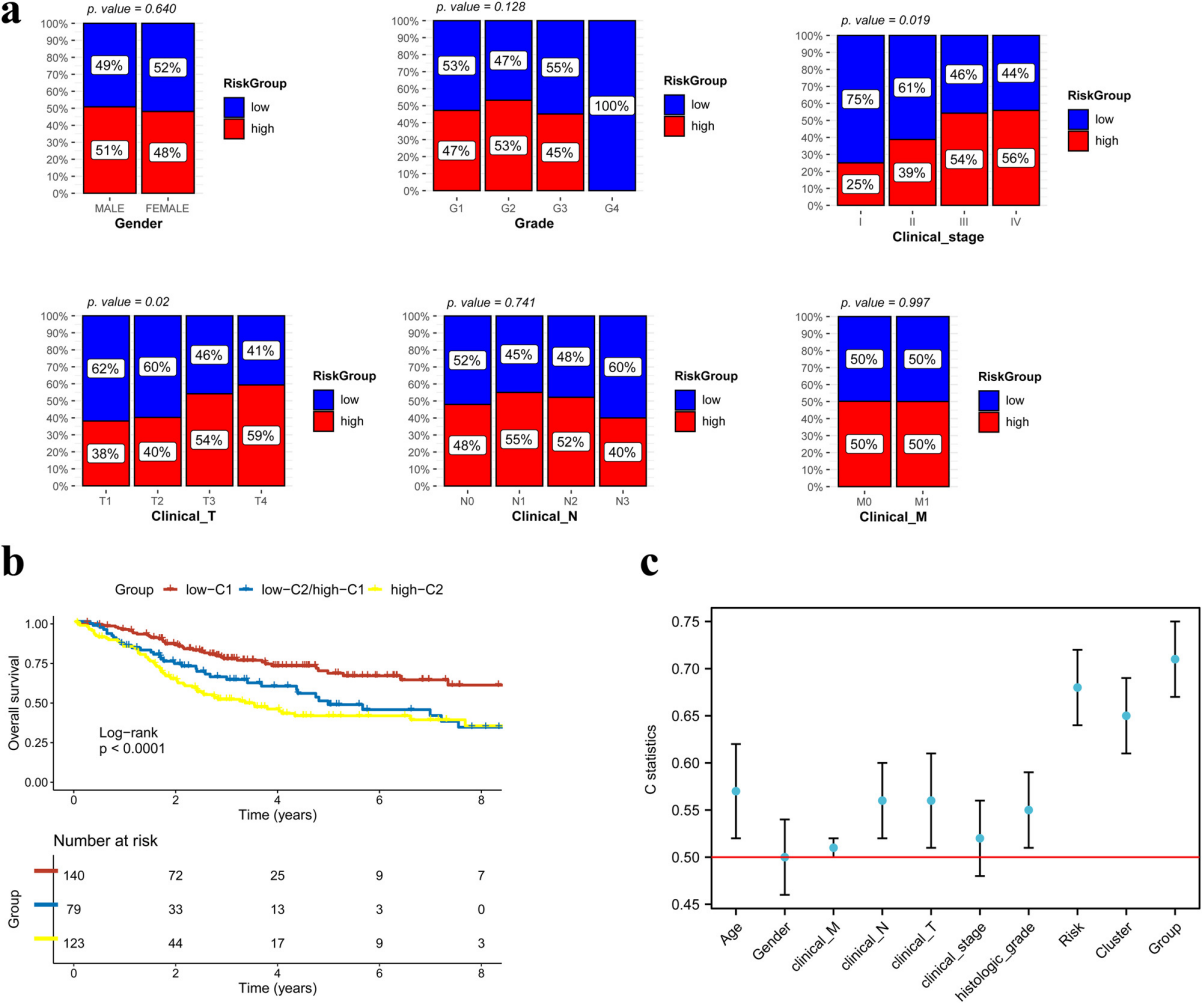
Clinical value of subtype and risk-score groups. (a) The clinical predictive ability of the risk score in gender, grade, tumor stage, and TNM. (b) Kaplan–Meier curve among the three new groups: low-C1 (low-risk group and cluster 1), low-C2/high C1 ([low-risk group and cluster 2] or [high-risk group and cluster 1]), high-C2 (high-risk group and cluster 2). (c) C-index error in all prognostic clinical features, subtype, risk score, and the new conjoint feature.

Clinical analysis showed that the high- and low-risk groups exhibited significant differences in clinical T category and stage (Figure 7a), demonstrating that the risk model may be able to predict the clinical features of the patients.

The two subtype groups and the two risk groups were highly correlated with the survival of patients with OCSS. Thus, we further divided all samples into three groups (low-C1, low-C2/high-C1, and high-C2), and analyzed their survival status using the Kaplan–Meier curve method (Figure 7b). The results showed that the high-C2 and low-C1 groups had the least and most favorable survival outcomes, respectively. The C-index of clinical factors (such as age, sex, TNM stage, and grade) and subtype, risk, and generated groups were input into the analysis (Figure 7c). The C-index of the generated groups was the highest (>0.7), indicating the most effective predictive ability.

## 4 Discussion

The immune and stromal scores in tumors were higher than those in normal tissue, indicating that OSCC is highly related to the immune microenvironment, which is consistent with the results of previous studies [25,26]. Bioinformatics has emerged as an effective approach for identifying molecules that are crucial in diseases. CIBERSORT has been successfully used to estimate immune cell infiltration in diverse diseases [27,28,29]. WGCNA is the most valuable tool for identifying and screening crucial gene modules and genes for cancer by constructing co-expression gene networks [12,30,31]. In this study, we conducted WGCNA analysis to identify immune-related genes in OSCC, and CIBERSORT to identify the infiltration level of immune cells. The Limma package was used to identify DEGs between tumor and normal tissues. In total, 3,783 DEGs were identified. Among the 3,783 DEGs, 221 were identified after WGCNA analysis as significantly related to immune characteristics (via immune and stromal scores). The function of these genes has been suggested to be associated with the immune response in this study.

In total, 34 prognostic genes were identified as prognosis-related genes in OSCC. The 34 genes were grouped into two subtypes, and the Kaplan–Meier curve suggested that patients in cluster 2 had a less favorable survival outcome than those in cluster 1. Further analysis showed lower infiltration levels of immune cells, such as CD8+ T-cells, regulatory T-cells, and M2 macrophages, in cluster 2, which might explain the less favorable survival outcome. Improved OS of patients with OSCC is associated with high CD8+ T-cell infiltration levels [7]. Zhou et al. also suggested the prognostic value of CD8+ T-cells [8]. Moreover, regulatory T-cells are also involved in the creation of an immunosuppressive environment in HNSCC [9] and OSCC [10]. These results provide an understanding of the prognostic abilities of the divided clusters.

In addition, 11 potential prognostic genes were included in the risk-score model after LASSO analysis. The risk score had independent predictive ability. Furthermore, Kaplan–Meier analysis showed that a high-risk score suggests a worse outcome for patients with OSCC. Differences in the immune cell infiltration landscape between the two risk groups may explain the differences in survival. CD8+ T-cell, regulatory T-cell, and M1 macrophage levels were significantly higher in the low-risk group than in the high-risk group. These differential immune cell infiltration landscapes may play a pivotal role in explaining the differential survival status between the two risk groups [7,8,10]. Macrophages are crucial for the prognosis of various types of cancer. M1 macrophages are generally considered to have pro-inflammatory and anti-tumor roles [25], and lower M1 macrophage levels may be detrimental to patients with OSCC. These results are consistent with those of previous studies showing that the level of cancer-related immune cells is a prognostic factor for OSCC [25,32]. Furthermore, the suitable AUC value of this model suggested its good performance in predicting 1, 3, and 5 year survival in OSCC. These results were verified using another GEO dataset. Together, these findings demonstrated the promising stable prognostic ability of the risk score.

Eleven potential prognostic genes were identified for the risk score. Previous studies have shown that, among these genes, *CTLA4*, *IGF2BP2*, and *TNFRSF4* are involved in cancer procession and affect the prognosis of patients with OSCC. *CTLA4* is extremely important for immune tolerance [33]. *CTLA4* is associated with recurrence- and metastasis-free survival in patients with OSCC [34]. A clinical analysis conducted for oral cancer showed that *CTLA4* can enhance the therapeutic efficacy of anti-PD-1 immunotherapy [35]. Furthermore, monoclonal anti-CTLA4 antibodies such as ipilimumab have been used for the treatment of advanced forms of various cancers like melanoma [36]. *IGF2BP2*, which is the receptor of N6-methyladenosine, is known as the insulin-like growth factor 2 mRNA-binding protein 2 [37]. Emerging evidence shows that *IGF2BP2* participates in the development and progression of cancers by communicating with different RNAs such as microRNAs, messenger RNAs, and long non-coding RNAs. Additionally, *IGF2BP2* is an independent prognostic factor for multiple cancer types [38]. In HNSCC tissues, *IGF2BP2* is highly expressed, and its high expression is associated with poor prognosis [37,39]. A recent study showed that *IGF2BP2* promotes HNSCC cell migration and invasion via the epithelial-mesenchymal transition process *in vitro*, and knockdown of *IGF2BP2* significantly inhibited lymphatic metastasis and lymphangiogenesis *in vivo* [40]. High *TNFRSF4* expression is associated with greater survival, suggesting a key role in HNSCC outcomes [41]. *KLHL6*, *HAO2*, and *OSR2* have been shown to be involved in the prognosis of other cancers. *KLHL6* expression levels in tumor tissue have prognostic value in gastric cancer [42]. The findings suggested that, in gastric cancer, *KLHL6* expression is lower in tumor tissues, and mice experiments revealed that the downregulation of KLHL6 expression also suppresses tumor growth. *HAO2* expression has a carcinostatic effect in hepatocellular carcinoma and prognostic ability [43]. *OSR2* has been identified as a potential biomarker for survival prognosis in muscle invasive bladder cancer [44], and its hypermethylation is a diagnostic marker in gastric cancer [45]. Although the roles of other genes involved in the prognostic model have been relatively less explored, some bioinformatics analyses have identified the prognostic role of some of these genes, such as *RTN4R* [46], *FCGBP* [47], and *FST* [48], in OSCC or other types of cancers. In general, the 11 genes tended to be important in the prognosis of OSCC. Here these genes were found to be related to immune cells and TIDE score. The TIDE score is considered the best predictor for ICI therapies [49]. A high TIDE score indicates a high potential for tumor immune evasion and low likelihood of benefiting from anti-PD-1/CTLA4 therapy. In this study, higher risk scores showed higher TIDE scores, further explaining the poor prognosis of patients with high-risk scores. More importantly, we investigated the clinical value of the risk score. The results showed that different risk groups were significantly different in clinical T category and stage, suggesting an application of the risk score in the clinic. Finally, to explore a more effective prediction method for patients with OSCC, a conjoint analysis combining the two subtypes and the two risk groups was conducted. The high-C2 group had the least favorable survival outcome, and the low-C1 group had the most favorable survival outcome, implying that the combined application of cluster subtypes and risk groups can predict the survival status of patients with OSCC. The C-index of the generated groups was the highest (>0.7), indicating that it had the most effective predictive ability.

In the present study, a prognostic subtype was identified, a predictive risk model for OSCC was established, and these were found to be effective in predicting the survival status of patients. As expected, the use of the conjoint analysis of the subtypes and risk scores showed the highest prognostic ability. We also analyzed the differential immune cell infiltration levels in various subtypes and risk groups, providing an understanding of the differential survival status to some extent. Finally, our findings suggest that the risk score is useful in predicting the immunotherapy response of patients with OSCC. However, this study has several limitations. First, the whole analysis conducted in TCGA possessed a small sample size of normal tissues. Although we conducted external validation of the GEO dataset, further verification of various datasets and clinical applications is still needed. Second, the roles of some of the potential prognostic genes involved in the risk score are still unknown. Thus, experimental IHC analysis and clinical validation are still needed.

## Supplementary Material

Supplementary Table

## References

[1] Solomon B, Young RJ, Rischin D. Head and neck squamous cell carcinoma: Genomics and emerging biomarkers for immunomodulatory cancer treatments. Semin Cancer Biol. 2018;52(Pt 2):228–40. 10.1016/j.semcancer.2018.01.008 29355614

[2] Braakhuis BJ, Leemans CR, Visser O. Incidence and survival trends of head and neck squamous cell carcinoma in the Netherlands between 1989 and 2011. Oral Oncol. 2014;50(7):670–5. 10.1016/j.oraloncology.2014.03.008 24735546

[3] Mes SW, Te Beest D, Poli T, Rossi S, Scheckenbach K, van Wieringen WN, et al. Prognostic modeling of oral cancer by gene profiles and clinicopathological co-variables. Oncotarget. 2017;8(35):59312–23. 10.18632/oncotarget.19576 PMC5601734 28938638

[4] Fuller CD, Wang SJ, Thomas CR Jr, Hoffman HT, Weber RS, Rosenthal DI. Conditional survival in head and neck squamous cell carcinoma: Results from the SEER dataset 1973–1998. Cancer. 2007;109(7):1331–43. 10.1002/cncr.22563 17326199

[5] Ribeiro IP, Esteves L, Santos A, Barroso L, Marques F, Caramelo F, et al. A seven-gene signature to predict the prognosis of oral squamous cell carcinoma. Oncogene. 2021;40(22):3859–69. 10.1038/s41388-021-01806-5 33972685

[6] Ferris RL, Blumenschein G, Jr, Fayette J, Guigay J, Colevas AD, Licitra L, et al. Nivolumab for recurrent squamous-cell carcinoma of the head and neck. N Engl J Med. 2016;375(19):1856–67. 10.1056/NEJMoa1602252 PMC5564292 27718784

[7] Shimizu S, Hiratsuka H, Koike K, Tsuchihashi K, Sonoda T, Ogi K, et al. Tumor-infiltrating CD8(+) T-cell density is an independent prognostic marker for oral squamous cell carcinoma. Cancer Med. 2019;8(1):80–93. 10.1002/cam4.1889 PMC6346233 30600646

[8] Zhou C, Wu Y, Jiang L, Li Z, Diao P, Wang D, et al. Density and location of CD3( +) and CD8( +) tumor-infiltrating lymphocytes correlate with prognosis of oral squamous cell carcinoma. J Oral Pathol Med. 2018;47(4):359–67. 10.1111/jop.12698 29469989

[9] Suzuki S, Ogawa T, Sano R, Takahara T, Inukai D, Akira S, et al. Immune-checkpoint molecules on regulatory T-cells as a potential therapeutic target in head and neck squamous cell cancers. Cancer Sci. 2020;111(6):1943–57. 10.1111/cas.14422 PMC7293074 32304268

[10] Liu S, Liu D, Li J, Zhang D, Chen Q. Regulatory T cells in oral squamous cell carcinoma. J Oral Pathol Med. 2016;45(9):635–9. 10.1111/jop.12445 27084296

[11] Cillo AR, Kürten CHL, Tabib T, Qi Z, Onkar S, Wang T, et al. Immune Landscape of Viral- and Carcinogen-Driven Head and Neck Cancer. Immunity. 2020;52(1):183–99.e9. 10.1016/j.immuni.2019.11.014 PMC7201194 31924475

[12] Cui Z, Bhandari R, Lei Q, Lu M, Zhang L, Zhang M, et al. Identification and exploration of novel macrophage M2-related biomarkers and potential therapeutic agents in endometriosis. Front Mol Biosci. 2021;8:656145. 10.3389/fmolb.2021.656145 PMC8290202 34295919

[13] Yan T, Zhu S, Zhu M, Wang C, Guo C. Integrative identification of hub genes associated with immune cells in atrial fibrillation using weighted gene correlation network analysis. Front Cardiovasc Med. 2020;7:631775. 10.3389/fcvm.2020.631775 PMC7859264 33553270

[14] Peng Y, Peng C, Fang Z, Chen G. Bioinformatics analysis identifies molecular markers regulating development and progression of endometriosis and potential therapeutic drugs. Front Genet. 2021;12:622683. 10.3389/fgene.2021.622683 PMC8372410 34421979

[15] Lohavanichbutr P, Méndez E, Holsinger FC, Rue TC, Zhang Y, Houck J, et al. A 13-gene signature prognostic of HPV-negative OSCC: Discovery and external validation. Clin Cancer Res. 2013;19(5):1197–203. 10.1158/1078-0432.CCR-12-2647 PMC3593802 23319825

[16] Hu D, Zhou M, Zhu X. Deciphering immune-associated genes to predict survival in clear cell renal cell cancer. Biomed Res Int. 2019;2019:2506843. 10.1155/2019/2506843 PMC6925759 31886185

[17] Ritchie ME, Phipson B, Wu D, Hu Y, Law CW, Shi W, et al. Limma powers differential expression analyses for RNA-sequencing and microarray studies. Nucleic Acids Res. 2015;43(7):e47. 10.1093/nar/gkv007 PMC4402510 25605792

[18] Wang L, Cao C, Ma Q, Zeng Q, Wang H, Cheng Z, et al. RNA-seq analyses of multiple meristems of soybean: Novel and alternative transcripts, evolutionary and functional implications. BMC Plant Biol. 2014;14:169. 10.1186/1471-2229-14-169 PMC4070088 24939556

[19] Langfelder P, Horvath S. WGCNA: An R package for weighted correlation network analysis. BMC Bioinforma. 2008;9(1):1–3. 10.1186/1471-2105-9-559 PMC2631488 19114008

[20] Huang da W, Sherman BT, Lempicki RA. Systematic and integrative analysis of large gene lists using DAVID bioinformatics resources. Nat Protoc. 2009;4(1):44–57. 10.1038/nprot.2008.211 19131956

[21] Rizvi AA, Karaesmen E, Morgan M, Preus L, Wang J, Sovic M, et al. gwasurvivr: An R package for genome-wide survival analysis. Bioinformatics. 2019;35(11):1968–70. 10.1093/bioinformatics/bty920 PMC7963072 30395168

[22] Zhang X, Ren L, Yan X, Shan Y, Liu L, Zhou J, et al. Identification of immune-related lncRNAs in periodontitis reveals regulation network of gene-lncRNA-pathway-immunocyte. Int Immunopharmacol. 2020;84:106600. 10.1016/j.intimp.2020.106600 32417654

[23] Tibshirani R. The LASSO method for variable selection in the Cox model. Stat Med. 1997;16(4):385–95. 10.1002/(sici)1097-0258(19970228)16:4<385::aid-sim380>3.0.co;2-3 9044528

[24] Jiang P, Gu S, Pan D, Fu J, Sahu A, Hu X, et al. Signatures of T cell dysfunction and exclusion predict cancer immunotherapy response. Nat Med. 2018;24(10):1550–8. 10.1038/s41591-018-0136-1 PMC6487502 30127393

[25] Alves AM, Diel LF, Lamers ML. Macrophages and prognosis of oral squamous cell carcinoma: A systematic review. J Oral Pathol Med. 2018;47(5):460–7. 10.1111/jop.12643 28940738

[26] Fraga M, Yáñez M, Sherman M, Llerena F, Hernandez M, Nourdin G, et al. Immunomodulation of T Helper cells by tumor microenvironment in oral cancer is associated with CCR8 expression and rapid membrane vitamin D signaling pathway. Front Immunol. 2021;12:643298. 10.3389/fimmu.2021.643298 PMC8137990 34025655

[27] Zhao Y, Zhang M, Pu H, Guo S, Zhang S, Wang Y. Prognostic implications of pan-cancer CMTM6 Expression and its relationship with the immune microenvironment. Front Oncol. 2020;10:585961. 10.3389/fonc.2020.585961 PMC7855963 33552963

[28] Kawada JI, Takeuchi S, Imai H, Okumura T, Horiba K, Suzuki T, et al. Immune cell infiltration landscapes in pediatric acute myocarditis analyzed by CIBERSORT. J Cardiol. 2021;77(2):174–8. 10.1016/j.jjcc.2020.08.004 32891480

[29] Kim Y, Kang JW, Kang J, Kwon EJ, Ha M, Kim YK, et al. Novel deep learning-based survival prediction for oral cancer by analyzing tumor-infiltrating lymphocyte profiles through CIBERSORT. Oncoimmunology. 2021;10(1):1904573. 10.1080/2162402X.2021.1904573 PMC8018482 33854823

[30] Zhao X, Zhang L, Wang J, Zhang M, Song Z, Ni B, et al. Identification of key biomarkers and immune infiltration in systemic lupus erythematosus by integrated bioinformatics analysis. J Transl Med. 2021;19(1):35. 10.1186/s12967-020-02698-x PMC7814551 33468161

[31] Xu M, Meng Y, Li Q, Charwudzi A, Qin H, Xiong S. Identification of biomarkers for early diagnosis of multiple myeloma by weighted gene co-expression network analysis and their clinical relevance. Hematology. 2022;27(1):322–31. 10.1080/16078454.2022.2046326 35231203

[32] Kikuchi M, Yamashita D, Hara S, Takebayashi S, Hamaguchi K, Mizuno K, et al. Clinical significance of tumor-associated immune cells in patients with oral squamous cell carcinoma. Head Neck. 2021;43(2):534–43. 10.1002/hed.26498 33029887

[33] Wong YK, Chang KW, Cheng CY, Liu CJ. Association of CTLA-4 gene polymorphism with oral squamous cell carcinoma. J Oral Pathol Med. 2006;35(1):51–4. 10.1111/j.1600-0714.2005.00377.x 16393254

[34] Koike K, Dehari H, Ogi K, Shimizu S, Nishiyama K, Sonoda T, et al. Prognostic value of FoxP3 and CTLA-4 expression in patients with oral squamous cell carcinoma. PLoS One. 2020;15(8):e0237465. 10.1371/journal.pone.0237465 PMC7423125 32785290

[35] Dorta-Estremera S, Hegde VL, Slay RB, Sun R, Yanamandra AV, Nicholas C, et al. Targeting interferon signaling and CTLA-4 enhance the therapeutic efficacy of anti-PD-1 immunotherapy in preclinical model of HPV(+) oral cancer. J Immunother Cancer. 2019;7(1):252. 10.1186/s40425-019-0728-4 PMC6749627 31533840

[36] Karimi A, Alilou S, Mirzaei HR. Adverse events following administration of anti-CTLA4 antibody ipilimumab. Front Oncol. 2021;11:624780. 10.3389/fonc.2021.624780 PMC7985548 33767992

[37] Chou CH, Chang CY, Lu HJ, Hsin MC, Chen MK, Huang HC, et al. IGF2BP2 polymorphisms are associated with clinical characteristics and development of oral cancer. Int J Mol Sci. 2020;21(16):5662. 10.3390/ijms21165662 PMC7460642 32784624

[38] Wang J, Chen L, Qiang P. The role of IGF2BP2, an m6A reader gene, in human metabolic diseases and cancers. Cancer Cell Int. 2021;21(1):99. 10.1186/s12935-021-01799-x PMC7876817 33568150

[39] Deng X, Jiang Q, Liu Z, Chen W. Clinical significance of an m6A reader gene, IGF2BP2, in head and neck squamous cell carcinoma. Front Mol Biosci. 2020;7:68. 10.3389/fmolb.2020.00068 PMC7193208 32391379

[40] Yu D, Pan M, Li Y, Lu T, Wang Z, Liu C, et al. RNA N6-methyladenosine reader IGF2BP2 promotes lymphatic metastasis and epithelial-mesenchymal transition of head and neck squamous carcinoma cells via stabilizing slug mRNA in an m6A-dependent manner. J Exp Clin Canc Res. 2022;41(1):6. 10.1186/s13046-021-02212-1 PMC8722037 34980207

[41] Qi Z, Liu Y, Mints M, Mullins R, Sample R, Law T, et al. Single-cell deconvolution of head and neck squamous cell carcinoma. Cancers. 2021;13(6):1230. 10.3390/cancers13061230 PMC7999850 33799782

[42] Deng J, Guo J, Ma G, Zhang H, Sun D, Hou Y, et al. Prognostic value of the cancer oncogene Kelch-like 6 in gastric cancer. Brit J Surg. 2017;104(13):1847–56. 10.1002/bjs.10628 29044464

[43] Mattu S, Fornari F, Quagliata L, Perra A, Angioni MM, Petrelli A, et al. The metabolic gene HAO2 is downregulated in hepatocellular carcinoma and predicts metastasis and poor survival. J Hepatol. 2016;64(4):891–8. 10.1016/j.jhep.2015.11.029 26658681

[44] Uysal D, Kowalewski KF, Kriegmair MC, Wirtz R, Popovic ZV, Erben P. A comprehensive molecular characterization of the 8q22.2 region reveals the prognostic relevance of OSR2 mRNA in muscle invasive bladder cancer. PLoS One. 2021;16(3):e0248342. 10.1371/journal.pone.0248342 PMC7954304 33711044

[45] Li WH, Zhou ZJ, Huang TH, Guo K, Chen W, Wang Y, et al. Detection of OSR2, VAV3, and PPFIA3 methylation in the serum of patients with gastric cancer. Dis Markers. 2016;2016:5780538. 10.1155/2016/5780538 PMC4838789 27143812

[46] Chen W, Liao L, Lai H, Yi X, Wang D. Identification of core biomarkers associated with pathogenesis and prognostic outcomes of laryngeal squamous-cell cancer using bioinformatics analysis. Eur Arch Otorhinolaryngol. 2020;277(5):1397–408. 10.1007/s00405-020-05856-5 32067095

[47] Chi LH, Wu ATH, Hsiao M, Li YJ. A transcriptomic analysis of head and neck squamous cell carcinomas for prognostic indications. J Pers Med. 2021;11(8):782. 10.3390/jpm11080782 PMC8399099 34442426

[48] Yang W, Zhou W, Zhao X, Wang X, Duan L, Li Y, et al. Prognostic biomarkers and therapeutic targets in oral squamous cell carcinoma: A study based on cross-database analysis. Hereditas. 2021;158(1):15. 10.1186/s41065-021-00181-1 PMC8066950 33892811

[49] Fan T, Liu Y, Liu H, Wang L, Tian H, Zheng Y, et al. Comprehensive analysis of a chemokine- and chemokine receptor family-based signature for patients with lung adenocarcinoma. Cancer Immunol Immun. 2021;70(12):3651–67. 10.1007/s00262-021-02944-1 PMC10992915 33977344

