# Supplementary material for: Prognostic signature related to the immune environment of oral squamous cell carcinoma
Source: Open Life Sci. 2022 Sep 14;17(1):1135–47. doi: 10.1515/biol-2022-0467 (PMC9482419; doi:10.1515/biol-2022-0467)
Supplement: Supplementary Table [file biol-2022-0467-sm.pdf]

Supplementary material

Table S1: Results of univariate Cox analysis

Gene	HR	95% CI	*p* value
CTLA4	0.8472	(0.76708–0.93574)	0.001076284
ICOS	0.8567	(0.77533–0.94659)	0.002381856
TNFRSF4	0.8158	(0.7136–0.93274)	0.002891276
KLHL6	0.8316	(0.73352–0.94269)	0.00394973
LAIR2	0.8499	(0.76005–0.95026)	0.004298983
HAO2	0.7287	(0.58242–0.91167)	0.005622394
OSR2	0.8146	(0.70439–0.94195)	0.005664879
ZFP42	1.0872	(1.0226–1.156)	0.007500052
RTN4R	1.2464	(1.0604–1.465)	0.007566125
FCGBP	0.9041	(0.83705–0.9766)	0.01041185
IGJ	0.9278	(0.87553–0.98316)	0.0112686
FCRL3	0.8895	(0.81171–0.97473)	0.01213491
ABCB1	0.8673	(0.77587–0.96942)	0.01218818
IGF2BP2	1.1934	(1.035–1.376)	0.01494907
P2RY14	0.8676	(0.77324–0.97341)	0.01556815
LMO2	0.8033	(0.6716–0.96085)	0.01652738
CELF2	0.8669	(0.76899–0.97736)	0.01957717
TTBK1	0.8520	(0.74453–0.97508)	0.01998243
TIGIT	0.8939	(0.81297–0.98291)	0.020564
KCNA2	0.8383	(0.72138–0.97409)	0.02129753
CD40LG	0.8887	(0.80375–0.98265)	0.02137698
HPGDS	0.8639	(0.76214–0.97933)	0.02222952
S100B	0.8737	(0.77737–0.98207)	0.02361318
IPCEF1	0.8666	(0.76483–0.98181)	0.02457394
PARM1	0.8820	(0.78921–0.98567)	0.02679978
CLEC10A	0.9004	(0.81917–0.98969)	0.02963816
CFP	0.8804	(0.78474–0.98768)	0.02991689
TESC	0.8544	(0.74115–0.98503)	0.0301736
GPR15	0.9114	(0.83639–0.99311)	0.0341858
FST	1.1147	(1.0079–1.2328)	0.03457256
FOXP3	0.9035	(0.82171–0.99334)	0.0359082
KLRB1	0.9022	(0.81656–0.99671)	0.04289174
NAPSB	0.9061	(0.82266–0.99791)	0.04524576

Table S1: *Continued*

Gene	HR	95% CI	*p* value
IL21R	0.9023	(0.81486–0.99922)	0.04827246
PHLDB2	1.1671	(0.99876–1.3639)	0.05184551
ARRB1	0.8471	(0.71581–1.0024)	0.05338778
TNFRSF8	0.8906	(0.79179–1.0017)	0.05347264
CX3CR1	0.9110	(0.82868–1.0014)	0.05358817
PAQR8	0.8688	(0.75196–1.0038)	0.05630439
ID2	0.8835	(0.77535–1.0067)	0.06286142
BCL2	0.8849	(0.77629–1.0087)	0.06719496
CXCL13	0.9248	(0.85046–1.0057)	0.06763589
LYZ	0.9291	(0.85625–1.0082)	0.07777823
ABCD2	0.9122	(0.82204–1.0121)	0.08319513
KLRF1	0.8899	(0.77938–1.0161)	0.08467872
HMGA2	1.0649	(0.99092–1.1444)	0.08694462
GNGT1	1.1121	(0.98352–1.2575)	0.09009423
FCER1A	0.9292	(0.85321–1.012)	0.09179141
HKDC1	1.0595	(0.99042–1.1334)	0.09296814
CCR2	0.9281	(0.84945–1.0141)	0.09889358
SDS	1.0951	(0.98228–1.221)	0.1014745
IL2RA	0.9103	(0.81299–1.0192)	0.1031429
GPR55	0.9095	(0.81131–1.0196)	0.1038505
ATP2A3	0.8972	(0.78704–1.0228)	0.1047574
LOC286002	0.8862	(0.76535–1.026)	0.1060741
PPM1K	0.8742	(0.74188–1.0301)	0.10838
GK	0.8796	(0.7513–1.0298)	0.1107574
SPANXC	1.0555	(0.98764–1.1279)	0.1111956
CHI3L2	0.9391	(0.86896–1.015)	0.1131397
TMEM71	0.9068	(0.80319–1.0238)	0.1141044
CCR8	0.9269	(0.84338–1.0188)	0.1154267
ALOX12P2	1.0908	(0.97718–1.2176)	0.1214768
SPNS3	0.9024	(0.79177–1.0284)	0.1235976
SELP	0.9458	(0.88061–1.0158)	0.1258717
ZNF671	0.8985	(0.78259–1.0315)	0.1285836
CALB1	1.0341	(0.98984–1.0804)	0.132843

(*Continued*)

Table S1: *Continued*

Gene	HR	95% CI	*p* value
LILRA3	0.9355	(0.85762–1.0205)	0.1331301
GYPC	0.9035	(0.79072–1.0323)	0.1356605
C10orf91	1.0905	(0.97308–1.222)	0.1361387
TNFRSF9	0.9209	(0.82514–1.0279)	0.1417496
ZFYVE28	0.8882	(0.75804–1.0408)	0.1428498
LHX5	1.0748	(0.97575–1.1839)	0.1436643
MAN1C1	0.9118	(0.80358–1.0347)	0.1523299
IL4I1	0.9144	(0.80902–1.0336)	0.1523416
FAM46C	0.9328	(0.84808–1.0261)	0.1525399
PLAC1	1.0638	(0.97722–1.1582)	0.1532123
ITM2A	0.9235	(0.82782–1.0302)	0.1535158
MLANA	0.8839	(0.74432–1.0497)	0.1595572
NWD1	0.9355	(0.85096–1.0284)	0.1672589
LRRK2	0.9362	(0.85244–1.0281)	0.1676304
MMP12	0.9413	(0.86342–1.0261)	0.1693331
GFI1	0.9194	(0.81538–1.0367)	0.1700253
CCDC69	0.9069	(0.78704–1.0451)	0.1769286
CYSLTR1	0.9261	(0.82671–1.0375)	0.1853941
GRAMD3	0.8745	(0.71658–1.0671)	0.186642
IL22RA2	0.9417	(0.86118–1.0297)	0.1877878
NICN1	0.8523	(0.67175–1.0815)	0.1884284
PLD4	0.9396	(0.85591–1.0314)	0.1900104
LCN10	0.9163	(0.80286–1.0458)	0.1951984
REEP6	1.0986	(0.95272–1.2667)	0.1958495
COX6B2	0.9409	(0.85729–1.0326)	0.1988436
VAX1	1.0550	(0.97142–1.1458)	0.2035931
APPL1	0.8533	(0.66573–1.0937)	0.2103171
ALDH2	0.9186	(0.8023–1.0518)	0.219241
KCNK5	0.9142	(0.79144–1.056)	0.2228322
ARTN	1.0717	(0.95776–1.1992)	0.2273062
TXNIP	0.9326	(0.83211–1.0452)	0.2300697
PTPRH	1.0620	(0.96183–1.1726)	0.2340011
FXYD2	0.9411	(0.85126–1.0405)	0.2360484
SLC2A1	1.0880	(0.94585–1.2515)	0.2377739
RNF125	0.9157	(0.79023–1.0611)	0.2413203
GGTA1	0.9288	(0.8208–1.0511)	0.2418015
SLC7A11	1.0501	(0.9673–1.14)	0.2433909
C3	1.0595	(0.96126–1.1678)	0.2442576
SLAMF8	0.9316	(0.82618–1.0504)	0.2470635
IFI30	0.9123	(0.78045–1.0665)	0.2494405

Table S1: *Continued*

Gene	HR	95% CI	*p* value
PM20D1	0.9011	(0.75228–1.0794)	0.2582076
CLEC4E	0.9431	(0.85158–1.0445)	0.2610905
SLC14A1	0.9322	(0.82363–1.0552)	0.2667935
DNASE1L3	0.9551	(0.87833–1.0386)	0.2825135
CD1C	0.9425	(0.84519–1.0509)	0.2863476
KCNMB1	0.9154	(0.77733–1.0781)	0.2895897
CCL7	0.9466	(0.85522–1.0478)	0.2898822
PLCL2	0.9376	(0.83222–1.0564)	0.2900617
CD200R1	0.9420	(0.84226–1.0536)	0.2955626
CLDN23	0.9340	(0.81957–1.0643)	0.3053107
ST6GAL1	0.9466	(0.85056–1.0536)	0.3154228
CD1E	0.9567	(0.8774–1.0432)	0.3161512
GBX2	1.0522	(0.94967–1.1657)	0.3308927
PRKAR2B	0.9403	(0.8304–1.0648)	0.3320981
CCL18	0.9613	(0.88736–1.0414)	0.3339445
KPNA7	0.9459	(0.84158–1.0631)	0.3506618
MSRA	0.9224	(0.77746–1.0942)	0.3539161
HCG4	1.0436	(0.95269–1.1432)	0.3586908
SLC11A1	1.0637	(0.93116–1.2151)	0.3630856
SIGLEC12	0.9650	(0.89273–1.043)	0.368894
WNT7B	1.0783	(0.91123–1.276)	0.3800728
APOC1	1.0369	(0.95401–1.1271)	0.3937663
FGL2	0.9586	(0.86965–1.0566)	0.3943059
ATP1A3	1.0487	(0.9396–1.1706)	0.3959962
C5orf38	1.0419	(0.94521–1.1485)	0.4087114
HTRA4	0.9519	(0.84424–1.0733)	0.4206302
SLC39A4	1.0623	(0.91622–1.2316)	0.4234456
NCF2	0.9341	(0.78986–1.1047)	0.4256235
FPR2	0.9605	(0.86981–1.0606)	0.4256258
ITGAX	0.9535	(0.84589–1.0749)	0.43616
GAST	1.0305	(0.95525–1.1116)	0.4377454
MYO7A	0.9418	(0.80854–1.0969)	0.4406301
DUSP5P	0.9581	(0.85217–1.0771)	0.4736193
PCSK9	1.0341	(0.94163–1.1357)	0.482752
SPANXB2	1.0239	(0.95852–1.0937)	0.4833799
LILRB4	0.9693	(0.8872–1.0589)	0.4891854
SIDT1	0.9658	(0.87516–1.0659)	0.4894845
MAGEC2	0.9795	(0.92309–1.0393)	0.4925965
CCL19	0.9784	(0.91827–1.0424)	0.4993927
CD80	0.9605	(0.85342–1.081)	0.5039803

(*Continued*)

Table S1: *Continued*

Gene	HR	95% CI	*p* value
MSLN	0.9835	(0.93604–1.0333)	0.5086269
FBXL17	0.9297	(0.74745–1.1564)	0.5126726
PLA2G7	0.9629	(0.85925–1.079)	0.5146165
HOXD11	1.0273	(0.94697–1.1145)	0.5162401
HNMT	0.9580	(0.84113–1.0911)	0.5182247
NMB	0.9621	(0.85472–1.0831)	0.5228875
TRPM2	1.0386	(0.92445–1.1669)	0.5236421
BFSP1	0.9548	(0.82833–1.1007)	0.523905
MAGEC1	0.9822	(0.92874–1.0388)	0.5297677
CCL2	0.9670	(0.8674–1.0781)	0.5453105
GSTO2	0.9734	(0.89129–1.063)	0.5482436
CCL23	0.9604	(0.84159–1.096)	0.548673
TMC7	1.0488	(0.89751–1.2255)	0.5490911
SUN3	1.0280	(0.93881–1.1256)	0.5514192
CD207	0.9780	(0.90853–1.0529)	0.5548707
SNX10	1.0474	(0.89501–1.2258)	0.5636964
SRMS	0.9697	(0.87007–1.0807)	0.5776114
CD300LF	0.9681	(0.8631–1.0858)	0.5791702
EMX1	1.0258	(0.93667–1.1234)	0.5830703
ADAMDEC1	0.9811	(0.91646–1.0504)	0.5839601
SP140	0.9684	(0.86074–1.0895)	0.5929155
APOE	1.0257	(0.93412–1.1263)	0.5948919
ASAH1	0.9363	(0.73145–1.1985)	0.6012106
MAP3K14	0.9492	(0.77983–1.1553)	0.6030025
ADAMTS20	0.9814	(0.91365–1.0541)	0.6064816
NAAA	0.9553	(0.79688–1.1451)	0.6207181
NTSR1	0.9823	(0.91394–1.0557)	0.626774
C2	0.9721	(0.86528–1.0922)	0.6340943
CD70	1.0203	(0.93719–1.1108)	0.6427566
CNTD2	0.9772	(0.88571–1.0781)	0.6455576
TREM2	0.9754	(0.87312–1.0896)	0.6589687
APOC2	1.0202	(0.93271–1.1158)	0.6623943
SLAMF9	0.9813	(0.89946–1.0706)	0.67139
SPANXA2	1.0155	(0.94328–1.0933)	0.6823136
CAB39L	1.0427	(0.84522–1.2862)	0.6965199
EMR2	1.0295	(0.88877–1.1926)	0.6979992
PLA1A	0.9807	(0.88806–1.083)	0.7004292
IL33	0.9866	(0.9198–1.0582)	0.7049913

Table S1: *Continued*

Gene	HR	95% CI	*p* value
ATP1B3	0.9631	(0.78911–1.1754)	0.7112417
CCNG1	1.0472	(0.81659–1.343)	0.7162186
GALNTL4	0.9668	(0.80428–1.1622)	0.7194693
ENPP4	1.0170	(0.92602–1.117)	0.7240736
NCOA7	1.0344	(0.85731–1.248)	0.7242214
N4BP2L1	0.9786	(0.86152–1.1115)	0.7387541
HK3	0.9841	(0.89206–1.0856)	0.7490704
SLC26A4	1.0229	(0.88782–1.1785)	0.7541122
XCL1	0.9839	(0.88573–1.0929)	0.7614149
ATP8A1	0.9840	(0.88591–1.0929)	0.7627756
C20orf195	0.9813	(0.86301–1.1159)	0.7737156
ST8SIA6	0.9823	(0.86841–1.111)	0.7758727
SLC6A12	0.9841	(0.8771–1.1041)	0.7843769
TSKS	1.0082	(0.94537–1.0751)	0.804338
CARD11	1.0121	(0.91624–1.118)	0.8124296
ICAM1	1.0169	(0.88248–1.1719)	0.8164686
TNIP3	0.9898	(0.9057–1.0816)	0.8202528
CISH	0.9761	(0.78592–1.2124)	0.827196
SNAI3	0.9849	(0.852–1.1386)	0.837211
CX3CL1	1.0083	(0.92346–1.101)	0.8532569
PKIB	1.0122	(0.8898–1.1514)	0.8540843
CLEC12A	1.0086	(0.91332–1.1139)	0.8650552
FCGR2A	1.0097	(0.88341–1.1539)	0.8878567
TMEM150B	1.0074	(0.89921–1.1287)	0.8983805
C21orf125	0.9911	(0.86407–1.1368)	0.8984503
TREM1	1.0052	(0.91722–1.1017)	0.9110345
P2RY12	1.0051	(0.90667–1.1142)	0.9232399
MGAT4A	0.9933	(0.86348–1.1425)	0.9245319
KMO	1.0058	(0.88719–1.1404)	0.9275518
NCRNA00174	0.9946	(0.8838–1.1194)	0.9288894
EIF4E3	0.9941	(0.82834–1.1931)	0.9494069
CXCL2	0.9970	(0.90578–1.0975)	0.9516572
FCHO1	1.0035	(0.89467–1.1256)	0.9523734
TGM4	1.0032	(0.89147–1.129)	0.9575298
C9orf66	1.0034	(0.8681–1.1598)	0.963364
AKD1	1.0031	(0.84946–1.1846)	0.9704776
FCGR3A	1.0012	(0.90616–1.1063)	0.9806327

Table S2: Results of LASSO analysis

Gene	LASSO coefficient	Gene	LASSO coefficient
CTLA4	−0.0215	KCNA2	−0.0023
ICOS	.	CD40LG	.
TNFRSF4	−0.0677	HPGDS	.
KLHL6	−0.0487	S100B	.
LAIR2	.	IPCEF1	.
HAO2	−0.0878	PARM1	.
OSR2	−0.1814	CLEC10A	.
ZFP42	0.0432	CFP	.
RTN4R	0.0803	TESC	
FCGBP	−0.0189	GPR15	.
IGJ	.	FST	0.037
FCRL3	.	FOXP3	.
ABCB1	.	KLRB1	.
IGF2BP2	0.0513	NAPSB	.
P2RY14	.	IL21R	.
LMO2	.		
CELF2	.		
TTBK1	.		
TIGIT	.		
